# Supplementary figures and images for: Meiosis reveals the early steps in the evolution of a neo-XY sex chromosome pair in the African pygmy mouse Mus minutoides
Source: PLoS Genet. 2020 Nov 12;16(11):e1008959. doi: 10.1371/journal.pgen.1008959 (PMC7685469; doi:10.1371/journal.pgen.1008959)

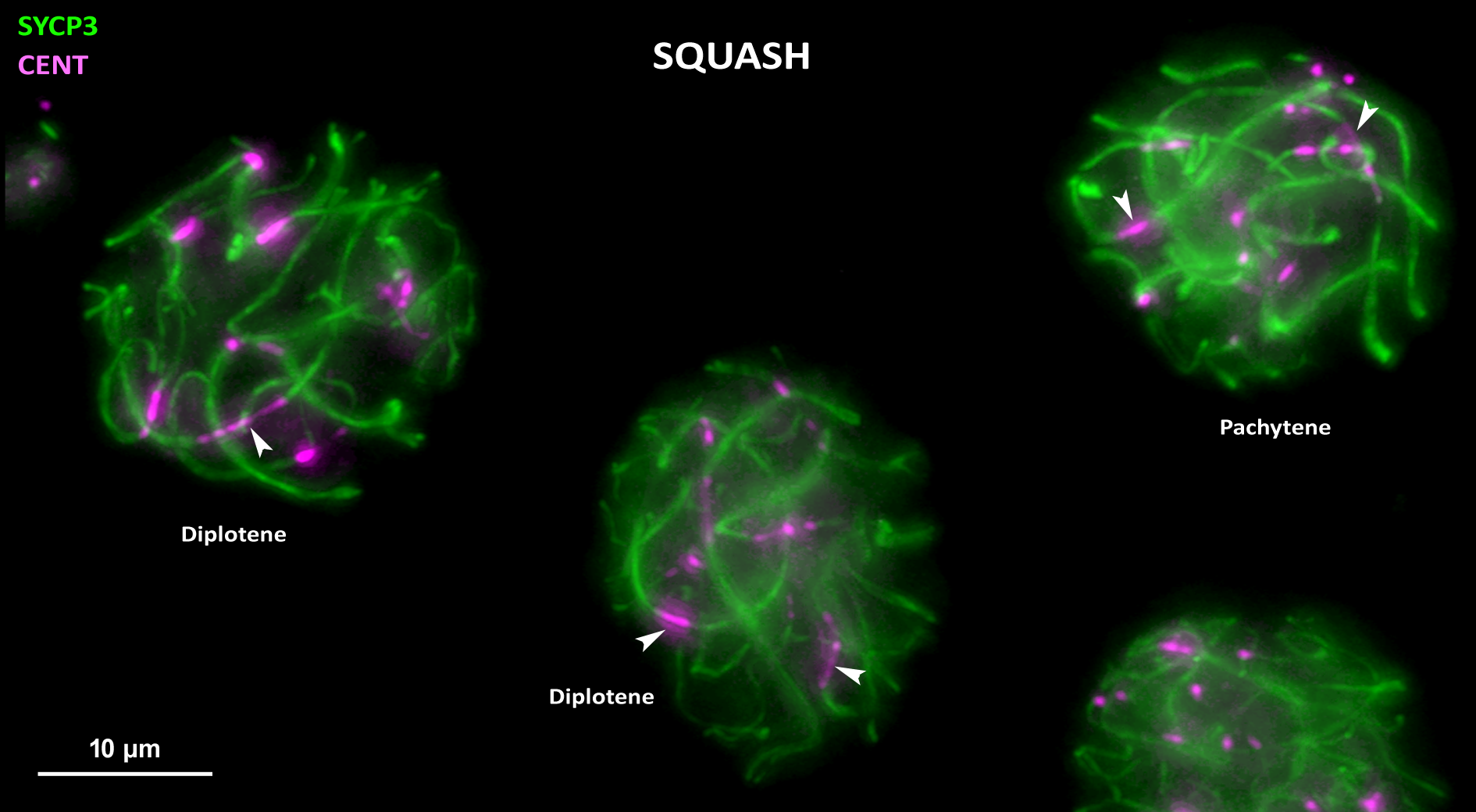

Supplement: S1 Fig — Squashed M. minutoides spermatocytes labelled with antibodies against SYCP3 (green) and centromeres (magenta). The stretching of centromeres (arrowheads) in pachytene and diplotene nuclei is observed clearly with this technique, which preserves the three-dimensional organization of the nucleus. Bar (TIF) [file pgen.1008959.s001.tif]

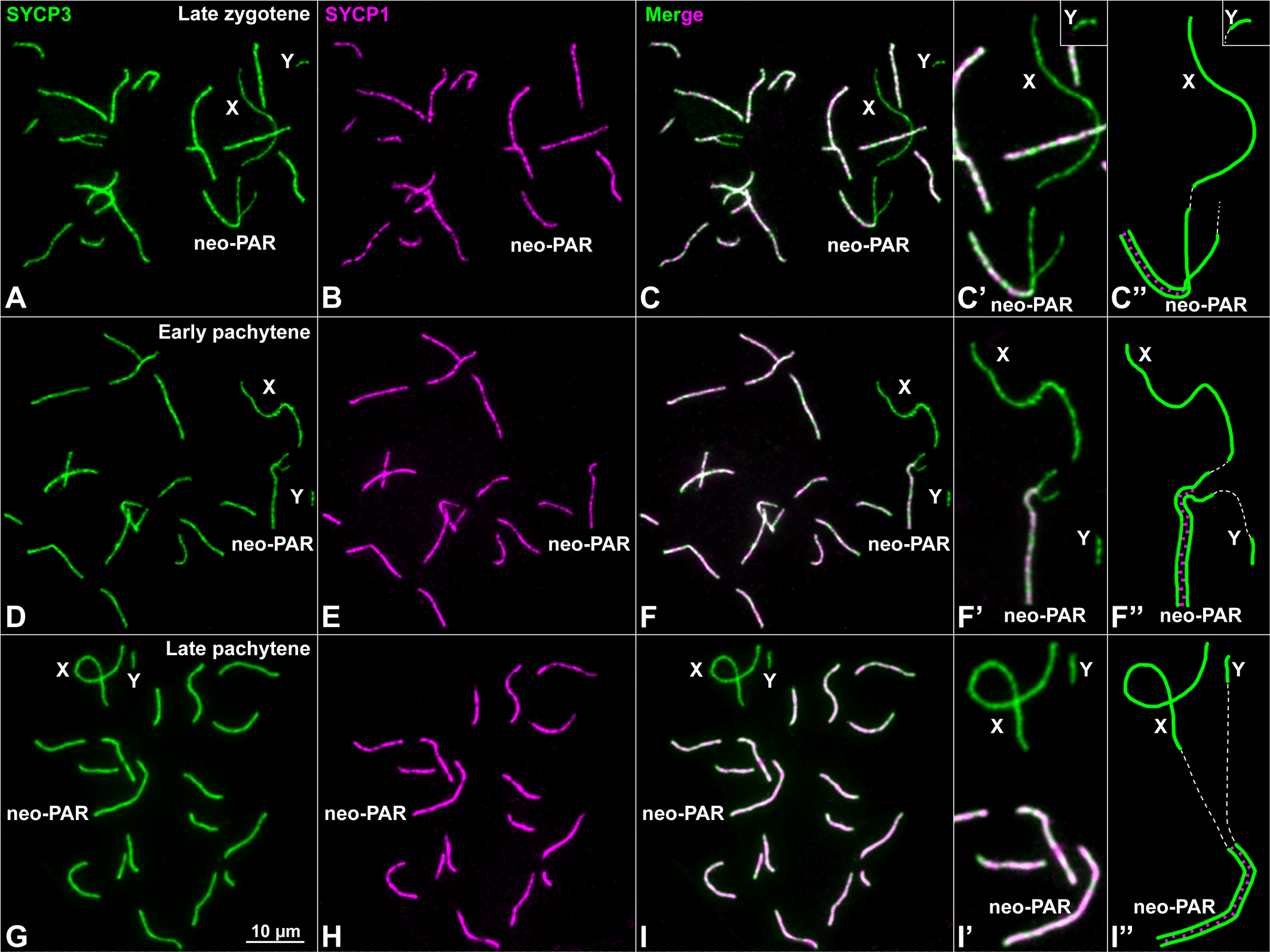

Supplement: S2 Fig — Synapsis in M. minutoides. Spread spermatocytes at different stages of prophase-I labelled with antibodies against SYCP3 (green) and SYCP1 (magenta). The heterologous segments of the sex chromosomes (X, Y) and the neo-PAR are indicated. A-C”. Late zygotene. Most autosomes have completed synapsis. SYCP1 and SYPC3 colocalize along the synapsed regions (yellow in C), while only SYCP3 is detectable along unsynapsed ones. C’. An enlarged view of the sex chromosomes shown in C. C”. Schematic representation of sex chromosome synapsis. LEs are represented in green, TF as short magenta lines and centromeres as white dashed lines. D-F”. Early pachytene. Autosomes are fully synapsed but the neo-PAR of the sex chromosomes has not yet completed synapsis. G-I”. Late pachytene. The neo-PAR is completely synapsed, but the non-homologous segments of the X and Y chromosomes remain unsynapsed and negative for SYCP1. (TIF) [file pgen.1008959.s002.tif]

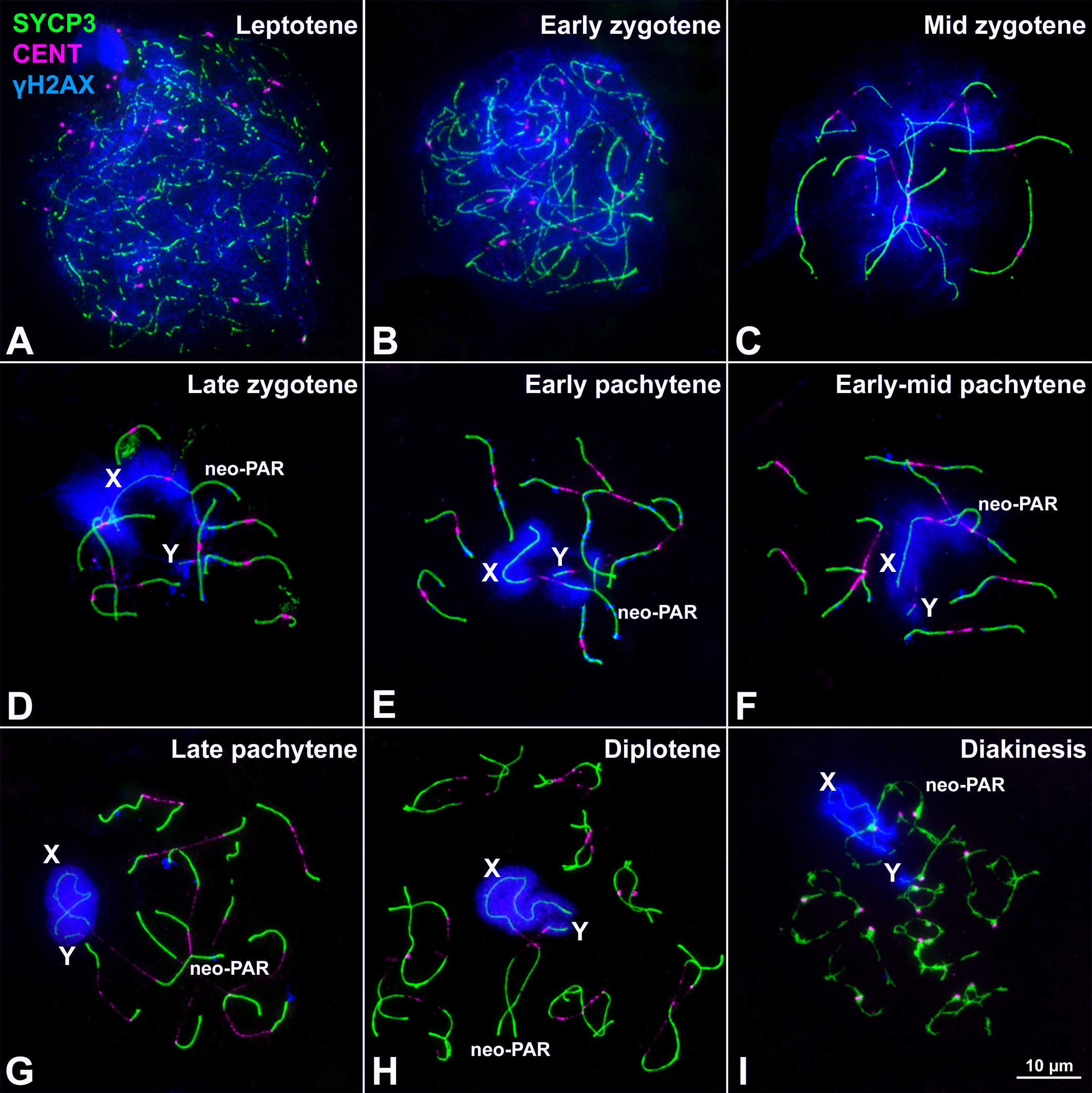

Supplement: S3 Fig — Spread spermatocytes at different stages of prophase-I labelled with antibodies against SYCP3 (green), centromeres (magenta) and γH2AX (blue). Sex chromosomes (X, Y) and the neo-PAR are indicated. A. Leptotene. SYPC3 forms short filaments, while γH2AX occupies the whole nucleus. B-D. Zygotene. SYCP3 forms thick filaments along the chromosomal regions that have completed synapsis. At early zygotene, the filaments are short, but elongate as synapsis proceeds. The γH2AX signal decreases during zygotene progression and, by late zygotene, only remains as a large focus on the unsynapsed regions of some autosomes and the sex chromosomes. Some small foci are also visible over synapsed autosomes. E-G. Pachytene. Autosomes have completed synapsis, but weak γH2AX signals can still be detected on them, mainly during early and mid pachytene. The neo-PAR of the sex chromosomes still remains partially unsynapsed at the transition from early to mid pachytene. During these stages, both the non-homologous segments and a portion of the neo-PAR are intensely labelled by γH2AX. By late pachytene, the neo-PAR appears completely synapsed. The non-homologous segments remain unsynapsed, though they associate with each other forming a sex body. H. Diplotene. The chromosomes initiate desynapsis and homologs remain associated at specific locations, likely chiasmata. γH2AX is observed only on the non-homologous segments of the sex chromosomes. I. Diakinesis. SYCP3 signal is irregular along the chromosomes and accumulates in some centromeric regions. γH2AX is only found on the non-homologous segments of the sex chromosomes. (TIF) [file pgen.1008959.s003.tif]

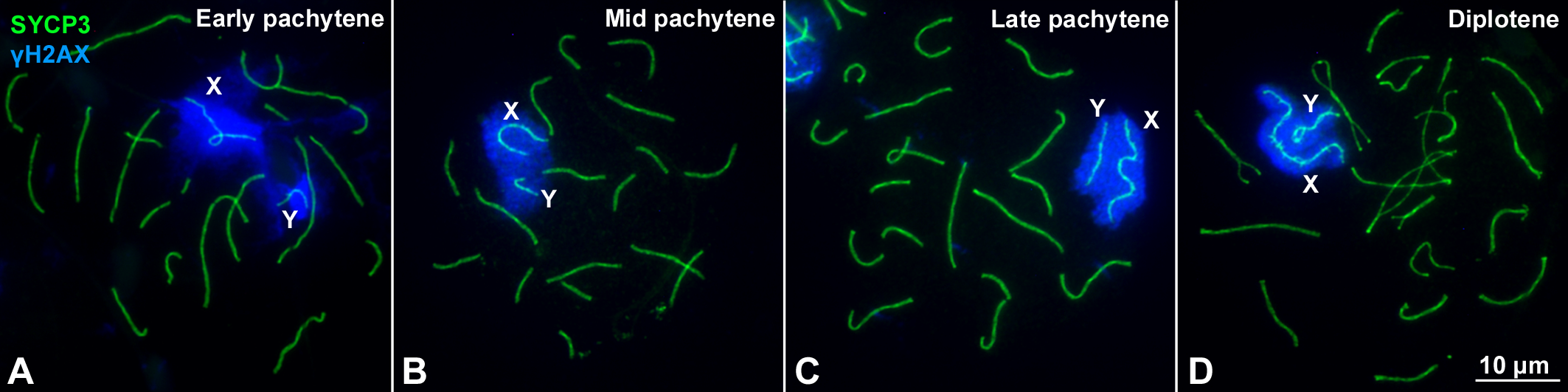

Supplement: S4 Fig — Spread spermatocytes at different stages of prophase-I labelled with antibodies against SYCP3 (green) and γH2AX (blue). Sex chromosomes (X, Y) are indicated. A. Early pachytene. All autosomes have completed synapsis, but some γH2AX signals can still be detected on some of them. Sex chromosomes still remain apart from each other and are intensely labelled by γH2AX, which has a fuzzy appearance. B. Mid pachytene. Sex chromosomes approach to each other and become associated in a single γH2AX signal, but no contact of their AEs is observed. C. Late pachytene. Sex chromosomes remain associated forming a typical sex body, intensely labeled with γH2AX. Their AEs show an irregular outline and usually bend. D. Diplotene. The autosomes initiate desynapsis while sex chromosomes remain associated. (TIF) [file pgen.1008959.s004.tif]

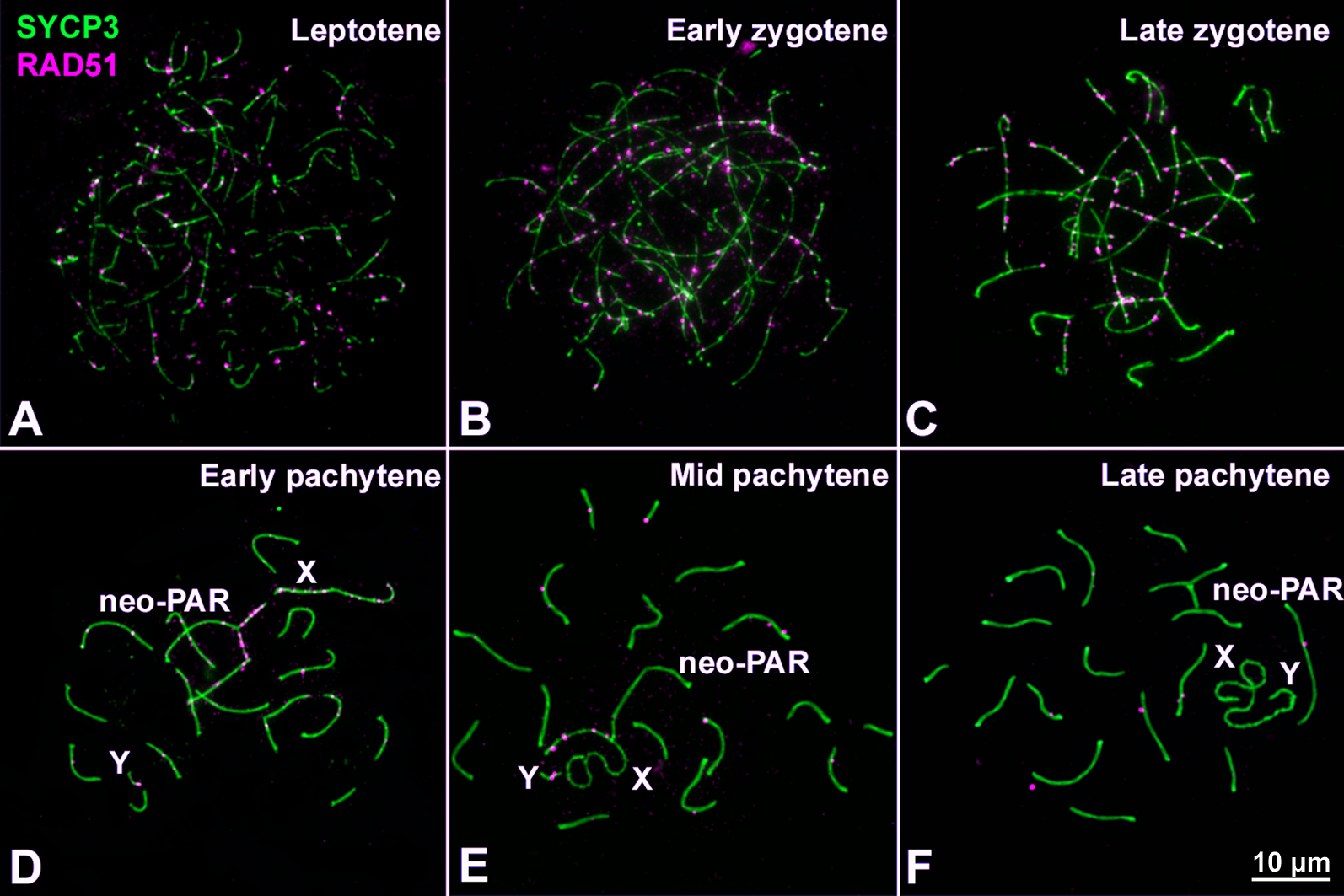

Supplement: S5 Fig — Spread spermatocytes at different stages of prophase-I labelled with antibodies against SYCP3 (magenta) and RAD51 (green). Sex chromosomes (X, Y) and the neo-PAR are indicated. A. Leptotene. RAD51 foci appear scattered over the nucleus, associated to the forming AEs. B. Early Zygotene. RAD51 is abundantly distributed over the unsynapsed AEs and the synapsed chromosomal regions. C. Late zygotene. The number of RAD51 foci decreases. These foci remain mainly associated to unsynapsed chromosomal regions. D. Early pachytene. RAD51 foci remain associated to unsynapsed chromosomes, mainly the proximal segment of the neo-PAR and the heterologous segments of the X and Y chromosomes (X, Y). E. Mid pachytene. Some RAD51 foci are present, mainly along the sex chromosomes (X, Y). F. Late pachytene. Only a few RAD51 are still detected with some autosomes, but not to sex chromosomes. (TIF) [file pgen.1008959.s005.tif]

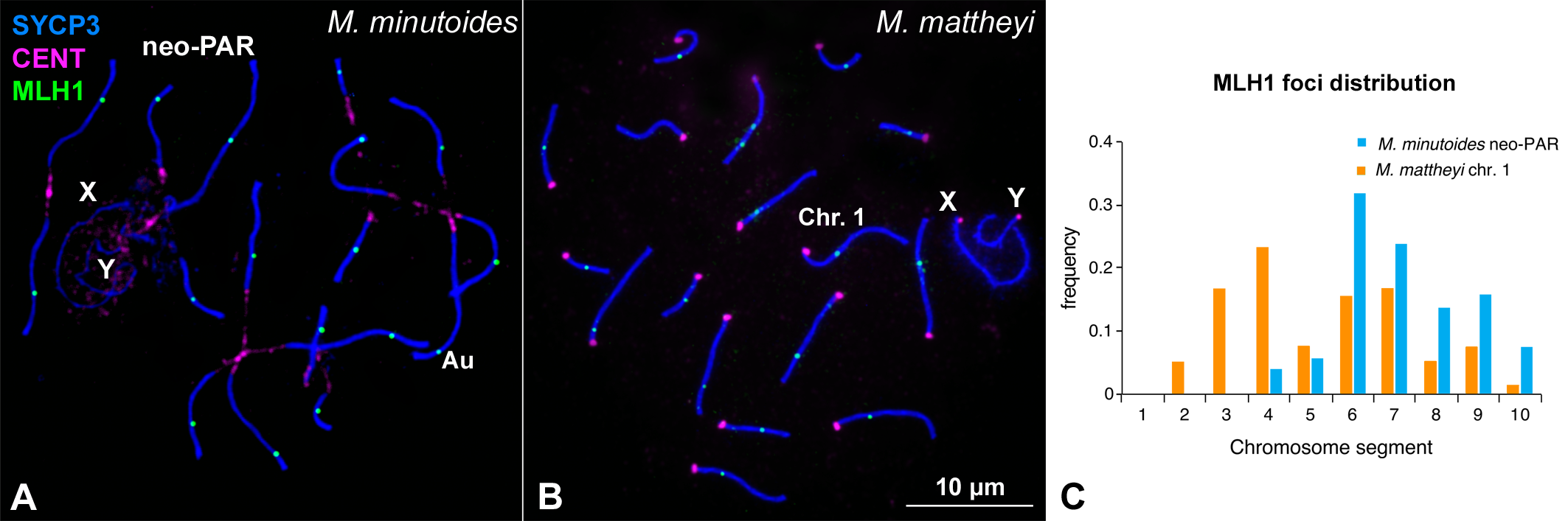

Supplement: S6 Fig — Spread spermatocytes of M. minutoides (A) and M. mattheyi (B) at pachytene labelled with antibodies against SYCP3 (blue), centromeres (magenta) and MLH1 (green). Sex chromosomes (X, Y) and the neo-PAR are indicated. Note the asynaptic behavior of sex chromosomes in M. mattheyi. C. Distribution of MLH1 foci along the neo-PAR in M. minutoides (blue) and the chromosome 1 in M. mattheyi (orange). 91 spermatocytes from three M. minutoides males and 72 spermatocytes from three M. mattheyi males were analyzed. The chromosomes have been divided into 10 equal segments from the centromere (1) to the distal telomere (10). (TIF) [file pgen.1008959.s006.tif]

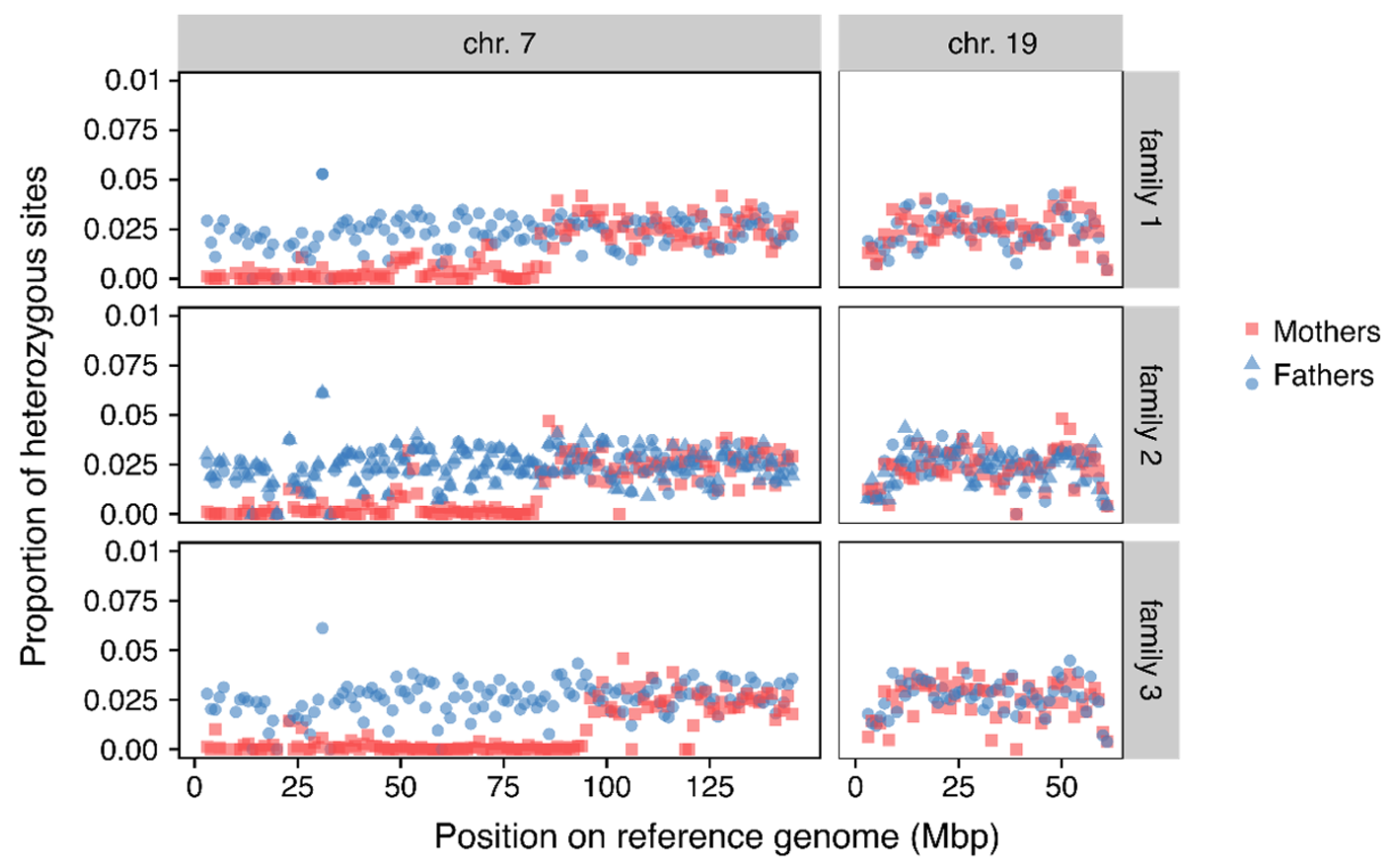

Supplement: S7 Fig — Proportion of heterozygous sites along the neo-sex chromosomes in mothers (magenta squares) and fathers (blue circles) based on the alignment position of RADtags to the M. musculus domesticus reference genome. Two fathers sired the offspring in family 2 and the heterozygosity for each is displayed (triangles instead of circles are used for one of the two fathers). Each point indicates the proportion of heterozygous sites averaged across a region of 1 megabase pairs (Mbp). Total number of RADtags mapping to chromosomes 7 and 19 for families 1, 2 and 3: 10,843; 10,833 and 10,228. (TIF) [file pgen.1008959.s007.tif]

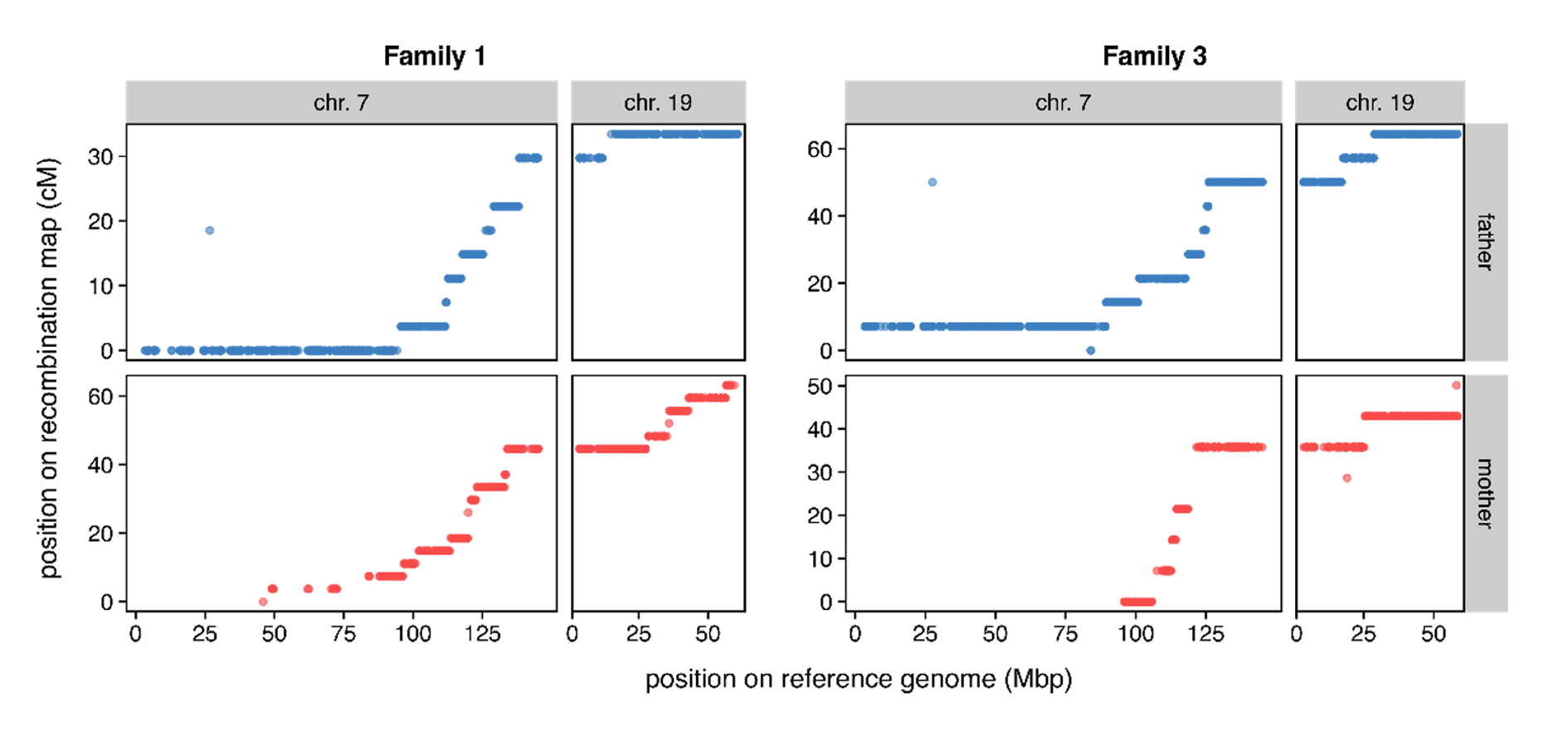

Supplement: S8 Fig — Each dot is a single marker (SNP); markers along the same horizontal line have segregated together within families. Breaks between lines of markers correspond to recombination events. cM: centi-Morgan, Mbp: megabase pairs. (TIF) [file pgen.1008959.s008.tif]
