# Supplementary material for: Meiosis reveals the early steps in the evolution of a neo-XY sex chromosome pair in the African pygmy mouse Mus minutoides
Source: PLoS Genet. 2020 Nov 12;16(11):e1008959. doi: 10.1371/journal.pgen.1008959 (PMC7685469; doi:10.1371/journal.pgen.1008959)
Supplement: S1 Table — (DOCX) [file pgen.1008959.s009.docx]

**S1 Table**

| Chromosome assigned | Male-limited tags | Fully sex-linked segregation pattern | | |
| --- | --- | --- | --- | --- |
|  |  | Family 1 | Family 2 | Family 3 |
| Chr 1 | **6** | 0 | 0 | **1** |
| Chr 2 | 0 | 0 | 0 | **1** |
| Chr 3 | **1** | 0 | 0 | 0 |
| Chr 4 | 0 | 0 | 0 | 0 |
| Chr 5 | **2** | 0 | 0 | 0 |
| Chr 6 | 0 | 0 | 0 | 0 |
| Chr 7 | **488** | **280** | **389** | **431** |
| Chr 8 | 0 | 0 | **1** | 0 |
| Chr 9 | **1** | 0 | 0 | 0 |
| Chr 10 | **1** | 0 | **1** | 0 |
| Chr 11 | **1** | 0 | 0 | 0 |
| Chr 12 | 0 | 0 | **1** | 0 |
| Chr 13 | 0 | 0 | **1** | **1** |
| Chr 14 | **2** | 0 | 0 | 0 |
| Chr 15 | 0 | **1** | **25** | **1** |
| Chr 16 | 0 | 0 | 0 | 0 |
| Chr 17 | **9** | **3** | **3** | **4** |
| Chr 18 | **1** | 0 | 0 | 0 |
| Chr 19 | 0 | 0 | 0 | 0 |
| Chr X | **3** | 0 | 0 | 0 |
| Chr Y | **23** | 0 | 0 | 0 |

**S1 Table.** Number of fully sex-linked tags that align to the different chromosomes of the *Mus musculus domesticus* reference genome.
