# Supplementary material for: Meiosis reveals the early steps in the evolution of a neo-XY sex chromosome pair in the African pygmy mouse Mus minutoides
Source: PLoS Genet. 2020 Nov 12;16(11):e1008959. doi: 10.1371/journal.pgen.1008959 (PMC7685469; doi:10.1371/journal.pgen.1008959)
Supplement: S2 Table — The two SNPs shown for each crossover event (SNP “before” and “after”) are the closest ones found on two adjacent blocks on the recombination map (a block is defined as group of SNPs with the same genetic position on the recombination map). (DOCX) [file pgen.1008959.s010.docx]

**S2 Table**

| family | parent | crossover  event | ID SNP before | ID SNP after | Chr. | Position of  SNP before | Position of  SNP after |
| --- | --- | --- | --- | --- | --- | --- | --- |
| family 1 | **father** | 1 | 76866_55 | 130733_49 | chr. 7 | 94267227 | 95593050 |
|  |  | 2 | 402771_86 | 195616_16 | chr. 7 | 111620423 | 111830934 |
|  |  | 3 | 238374_45 | 299543_65 | chr. 7 | 112060725 | 112663519 |
|  |  | 4 | 58200_82 | 57621_68 | chr. 7 | 117399976 | 117772451 |
|  |  | 5 | 306623_26 | 39768_21 | chr. 7 | 125305058 | 126174001 |
|  |  | 6 | 115891_85 | 186774_29 | chr. 7 | 128152403 | 129072804 |
|  |  | 7 | 279805_47 | 173462_85 | chr. 7 | 138160111 | 138392449 |
|  |  | 8 | 279805_47 | 173462_85 | chr. 7 | 138160111 | 138392449 |
|  |  | 9 | 165785_82 | 71316_15 | chr. 19 | 11839116 | 15037389 |
|  | **mother** | 1 | 90557_53 | 214335_68 | chr. 7 | 45923272 | 49015216 |
|  |  | 2 | 296068_47 | 298846_79 | chr. 7 | 72479031 | 83980649 |
|  |  | 3 | 81863_73 | 33406_91 | chr. 7 | 96225336 | 96681494 |
|  |  | 4 | 58314_42 | 157287_23 | chr. 7 | 100756993 | 102097635 |
|  |  | 5 | 140160_17 | 255732_30 | chr. 7 | 113142000 | 113615274 |
|  |  | 6 | 33468_9 | 260678_65 | chr. 7 | 119663035 | 119874621 |
|  |  | 7 | 33468_9 | 260678_65 | chr. 7 | 119663035 | 119874621 |
|  |  | 8 | 260678_65 | 11594_86 | chr. 7 | 119874621 | 120859858 |
|  |  | 9 | 361646_58 | 138162_74 | chr. 7 | 122609268 | 123097779 |
|  |  | 10 | 98115_23 | 277855_9 | chr. 7 | 132992466 | 133172673 |
|  |  | 11 | 406646_44 | 324769_41 | chr. 7 | 133398884 | 133980444 |
|  |  | 12 | 406646_44 | 324769_41 | chr. 7 | 133398884 | 133980444 |
|  |  | 13 | 30425_36 | 199542_74 | chr. 19 | 27446119 | 28451752 |
|  |  | 14 | 258291_86 | 164781_63 | chr. 19 | 35036993 | 35919361 |
|  |  | 15 | 164781_63 | 129528_59 | chr. 19 | 35919361 | 35980880 |
|  |  | 16 | 306422_40 | 415955_65 | chr. 19 | 42733271 | 42951393 |
|  |  | 17 | 388364_47 | 53367_22 | chr. 19 | 56336430 | 56574253 |
| family 3 | **father** | 1 | 243032_68 | 68284_67 | chr. 7 | 89217700 | 89457195 |
|  |  | 2 | 211306_89 | 33405_74 | chr. 7 | 100834226 | 101252283 |
|  |  | 3 | 10861_72 | 66449_49 | chr. 7 | 117703135 | 118755515 |
|  |  | 4 | 91267_69 | 14291_61 | chr. 7 | 123232373 | 124050039 |
|  |  | 5 | 276019_72 | 60419_12 | chr. 7 | 124994082 | 125333408 |
|  |  | 6 | 66955_39 | 156330_48 | chr. 7 | 125851650 | 125986888 |
|  |  | 7 | 457475_87 | 409111_35 | chr. 19 | 17176848 | 17625803 |
|  |  | 8 | 57623_58 | 441289_16 | chr. 19 | 28492391 | 28817150 |
|  | **mother** | 1 | 129436_27 | 336829_8 | chr. 7 | 105752333 | 107574345 |
|  |  | 2 | 194601_71 | 160780_34 | chr. 7 | 112682924 | 112967360 |
|  |  | 3 | 109550_43 | 46811_32 | chr. 7 | 114250716 | 114509038 |
|  |  | 4 | 371843_33 | 303262_88 | chr. 7 | 118623099 | 121643569 |
|  |  | 5 | 371843_33 | 303262_88 | chr. 7 | 118623099 | 121643569 |
|  |  | 6 | 100896_13 | 374702_53 | chr. 19 | 25108880 | 25439295 |

**S2 Table.** Alignment position of SNPs surrounding crossover events. The two SNPs shown for each crossover event (SNP “before” and “after”) are the closest ones found on two adjacent blocks on the recombination map (a block is defined as group of SNPs with the same genetic position on the recombination map).
